# Associated factors of home hospice care utilization by the terminally ill older adults: a mixed-methods study

**DOI:** 10.3389/fpubh.2025.1519712

**Published:** 2025-06-18

**Authors:** Jie Peng, Xiaoling Feng, Xiaoying Cao, Lichong Lai, Pengxin Dong, Haichen Wu, Yidan Chai, Ping Huang, Dongmei Hai, Caili Li, Yanfei Pan, Dejing Fan, Qini Pan, Shuyu Lu, Xiao Pan, Liyan Zhang, Pinyue Tao, HuiQiao Huang

**Affiliations:** The Second Affiliated Hospital of Guangxi Medical University, Nanning, China

**Keywords:** home hospice care, associated factors, Andersen model, terminally ill, older adults

## Abstract

**Objective:**

Given the discrepancy between the low utilization rate of hospice care services and the high willingness to choose hospice care, this study aims to identify the factors influencing the utilization among older adults. The findings will provide a foundation for interventions designed to enhance the growth of this service.

**Design:**

A mixed-methods study of convergent parallel designs.

**Methods:**

In the quantitative research phase, in January 2024, a convenience sampling method was used to recruit 350 terminally ill older individuals from three hospitals and three communities in Guangxi, China. Face-to-face surveys were conducted using a general information questionnaire, a Home Hospice Care Service Needs Questionnaire, and a Home Hospice Care Service Knowledge, Attitude, and Practice Scale. In the qualitative research phase, semi-structured interviews were conducted with 16 terminally ill older individuals to explore the barriers and facilitators to the utilization of home hospice care services.

**Results:**

A total of 332 (94.86%) older individuals completed the survey, and 45 (13.55%) accepted home hospice care. The identified barriers to utilization included high physiological, psychological, and spiritual needs, as well as families’ feelings of guilt. The facilitators included being of advanced age (≥80 years), having chronic diseases, not needing a caregiver, having high social needs and knowledge scores, and perceiving a significant economic burden.

**Conclusion:**

The utilization of home hospice care services by older adults is lower, which is influenced by both subjective and objective factors. The novel identification of ‘families’ guilty’ as a barrier underscores the need for targeted interventions that address emotional and psychological barriers within families. Policymakers should consider these factors when developing strategies to promote the development of home hospice care for older adults, ensuring that interventions are culturally sensitive and economically feasible.

## 1 Introduction

China’s aging process is accelerating, with the aged population expected to increase from 250 to 400 million by 2050, resulting in an aging rate exceeding 30% (1). As age increases, the risk of death increases and the ability to take care of themselves declines (2, 3). Among the older adults, 6.18 million were disabled, 82.34% were unable to look after themselves before they died (4, 5). Therefore, the quality of life for the older adult at the end of life is in urgent need of attention.

According to the report of the World Health Organization (6), globally, 25.7 million people need hospice care in the last year of life, with older adults accounting for over 69%. Due to the traditional Chinese cultural concept of ‘returning to one’s roots’ among Chinese older adults, approximately 87–89% of them pass away at home (7, 8). Home hospice care can improve the quality of life for older adults who die at home and their family members (9), reducing unnecessary hospitalizations and medical costs (10, 11). Therefore, studying home hospice care services for older adults is of great significance for improving the quality of life of older adults at the end of life.

According to the location of service, China’s hospice care service models include home hospice care and inpatient hospice care. Home hospice care mainly refers to terminally ill patients living at home, receiving basic life care from family members, and obtaining medical services or consultations from general practitioners and hospice care institutions to meet the needs of patients and their families (12). China has developed hospice care into a basic medical service, but its development started relatively late, with pilot programs beginning in 2017, and home hospice care services started even later (13). At present, China’s home hospice care service system is not mature and is unable to meet the needs of terminally ill older adults. Therefore, it is necessary to conduct more research. The willingness to utilize hospice care can promote its development (14). In South Korea, 88.9% of older adults at the end of life are willing to choose home hospice care (15); however, in China, 38.5% only (14). Compared with the global average, China’s utilization rate of hospice care services is even lower. Globally, 14% of people who need hospice care are able to receive such services, while in China, only 0.3% (15). In order to meet the needs of China’s large older population, it is necessary to explore the facilitators and barriers for older adults to utilize home hospice care services to promote the development of hospice care.

The development of hospice care in China is caught in a dilemma where there is a relatively high willingness but a low utilization rate of services (16). The reason for this dilemma is not just due to a lack of supply. In fact, the occupancy rate of hospice care beds is not high (15). Therefore, a systematic understanding of the factors influencing the utilization of home hospice care for terminally ill older adults could better facilitate its development. Existing research indicates that demographic characteristics, such as age (17, 18), health beliefs (19), economic income (20), perceived risk of illness (21), and attitudes toward death (22), influence the willingness or acceptance of hospice care services. It is evident that the factors influencing the utilization of hospice care encompass both objective and subjective elements. However, current research predominantly focuses on intentions, often employing either solely quantitative or qualitative methods, resulting in the study of factors influencing home hospice care utilization being somewhat limited. To comprehensively explore these factors and consider both objective conditions and subjective perceptions, it is essential to adopt a mixed-methods research approach.

The Andersen Model, a robust theoretical framework for analyzing the factors influencing the utilization of healthcare services, was proposed by American scholar Ronald Andersen (23). Therefore, guided by the Andersen Model, we employed a mixed-methods approach with a convergent parallel design to thoroughly investigate the associated factors of terminally ill older adults toward the utilization of home hospice care. This research aims to make up for the insufficiency of mixed-methods evidence in the research on factors influencing the utilization of home hospice care and provide actionable entry points to promote the development of home hospice care.

## 2 Theoretical framework

The Andersen Model posits that individual’s utilization healthcare services decided by the following three dimensions: predisposing factors, enabling factors, and need factors (23). Predisposing factors are characteristics that exist before an individual encounters a health problem, including demographic characteristics, social structure, health beliefs, and attitudes. Enabling factors are resources that facilitate or hinder access to healthcare services, including personal, family, and community resources. Need factors reflect the actual or perceived need for health services. Predisposing factors include demographic, social structure, and health belief variables. Enabling factors encompass personal, family, and social resources. Need factors involve the perception and evaluation of health status. After five revisions, the model can comprehensively explain health service utilization behavior (24).

## 3 Methods

### 3.1 Design

Our research team consists of 15 members, including two deputy chief physicians and five deputy chief nurses in the field of hospice care, seven graduate students, and one graduate advisor. Our research team was responsible for the design and implementation of the entire study. Due to the cultural background of taboo death in China (in Chinese culture, death is often considered a taboo topic, primarily due to the belief that discussing death brings bad luck or misfortune), this study adopted a mixed method of quantitative and qualitative research, which results can complement each other, enhancing the depth and breadth of the research on the associated factors of home hospice care for older adults. We conducted a convergent parallel design, meaning that both types of research were employed independently and simultaneously (25). The quantitative research was conducted using Paper-version questionnaires through one-on-one interviews, and the qualitative research was completed using semi-structured interviews. This study’s report refers to the Good Report of a Mixed-Methods Study (GRAMMS) (26).

### 3.2 Participants

In January 2024, a convenience sampling method was used to select 350 terminally ill older individuals from three hospitals and three communities in Guangxi, China, for the study. The surveyed hospitals and communities are all pilot sites for hospice care. For the qualitative study, the participants were recruited as follows: inclusion criteria included (1) older adult terminal patients (aged ≥60) or their primary caregivers who were deemed eligible for home hospice care by a physician and (2) those who provided informed consent to participate in the survey, and the exclusion criterion was individuals with communication difficulties. The inclusion criterion for the qualitative study was those who had completed the questionnaire and were willing to undergo a 20–30-min interview. According to logistic regression for sample size, at least 5–10 times the number of independent variables surveyed for the quantitative study is required and considered for a 10% sample loss (27). With 21 independent variables, the required sample size for the quantitative study was calculated to be 117–234 cases. The sample size for the qualitative study was based on the principle of saturation (28). Ultimately, a total of 332 (94.86%) terminally ill older individuals completed the questionnaire survey; 18 who failed to complete it were excluded, and 16 of them participated in qualitative interviews.

### 3.3 Research instruments

#### 3.3.1 General information

Our research team designed a survey questionnaire to collect general information about terminally ill older individuals after a comprehensive literature review and group discussions. Guided by the Andersen Model, general data are divided into the following three categories with detailed content as follows: (1) predisposing factors, including gender, age, ethnicity, whether suffering from chronic diseases, and awareness of home-based hospice care; (2) Enabling factors, including marital status, place of residence, education level, monthly personal income (Chinese Yuan), method of medical payment, living arrangement, and type of primary caregiver; (3) need factors include the activities of daily living (ADL) score. The Barthel Index Scoring Scale is used for scoring. The Barthel Index, designed by American Florence Mahoney and Dorothy Barthel (29), is applied clinically and is a widely used tool internationally. The total score is 100 points, with higher scores indicating greater self-care ability and less dependency; a score of 100 means no dependency, 61–99 points indicate mild dependency, 41–60 points indicate moderate dependency, and ≤40 points indicate severe dependency. The nutritional risk of older adults was measured using nutritional risk screening (NRS 2002), which was proposed by Danish scholar Kondrup in 2002 (30). The total score of NRS 2002 consists of three parts: nutritional status impairment score, disease severity score, and age score (over 70 years old plus 1 point). The total score is 0–7 points. If the patient’s score reaches or exceeds 3 points, it is considered that there is a nutritional risk, and nutritional support is required.

#### 3.3.2 Home hospice care service need scale

This scale was designed by our team, referring to China’s hospice care practice guidelines (31) and humanistic theories. The scale is divided into four dimensions: physiological needs (related to relieving symptoms, six items), psychological needs (related to helping patients cope with adverse emotional reactions, four items), social needs (related to individual social relations and support, seven items), and spiritual needs (related to patient’s beliefs, values and the pursuit of the meaning of life, four items), with a total of 21 items. The scoring uses a Likert 5-point scale as follows: Completely do not need (1 point), Occasionally need (2 points), Moderately need (3 points), Very much need (4 points), and Completely need (5 points). The total score is 21–105 points. The higher the total score, the greater the demand for home hospice care services. In our study, we confirmed that the scale has good reliability and validity when applied to terminally ill older adult individuals. Cronbach’s alpha is 0.949, and Scale-level Content Validity Index (S-CVI) is 0.923. See Appendix 1 for the scale.

#### 3.3.3 Knowledge, attitude, and behavior of home hospice care scale

Before the survey, our team developed the scale guided by the knowledge theory, belief theory, and action theory of the Behavioral Change Wheel (BCW) (32). The scale uses a Likert 5-point scoring system as follows: Strongly agree (5 points), Agree (4 points), Uncertain (3 points), Disagree (2 points), and Strongly disagree (1 point). To enhance the reliability of the survey results, seven items were reverse-scored. The total score is 29–145 points. The higher the total score, the better understanding of home hospice care. This scale has good reliability and validity, too, with a Cronbach’s alpha of 0.947 and an S-CVI of 0.941. See Appendix 2 for the scale.

#### 3.3.4 Interview outline

The interview outline was designed to focus on the psychological processes and associated factors involved in the use of home hospice care by older adults or their families, and it referenced relevant literature (33). The final version of the interview outline was modified and confirmed after conducting pre-interviews with two terminally ill older individuals to ensure the understandability and accessibility of the guide. The details are as follows: (1) How did you learn about home hospice care? What are your thoughts on it? (2) Why did you accept/refuse home hospice care services? (3) What barriers or difficulties have you encountered when receiving home hospice care services? (4) What are your needs for home hospice care services? (5) What suggestions do you have for the provision of home hospice care services?

## 4 Data collection

### 4.1 Quantitative data collection

Before the survey, uniform training was provided to the members of the research team. During the research process, the purpose and significance of the study were first introduced to the respondents. After obtaining their consent, the research team members conducted one-on-one interviews with each respondent using the questionnaire. After the interview, two researchers checked all the questionnaires, eliminated the questionnaires that were not completed and the answer rules, and then entered the data into the Excel table. Of them, 18 (5.14%) questionnaires were incomplete.

### 4.2 Qualitative data collection

Before the interview, staff from the hospice care institutions or hospitals communicated with the interviewees in advance. After the interviewees’ consent, two members of the research team conducted semi-structured interviews (one was responsible for the main interview, and the other was responsible for recording the interview dialogue, the interviewee’s expressions, and body language). The two members had received training in qualitative interviewing and participated in the collection and analysis of quantitative data. Hospitalized patients were interviewed in the hospital, while home-based patients were interviewed at their homes under the guidance of hospice care personnel. The interview environment was quiet and relaxed, with each interview lasting approximately 20–30 min. The interviewers asked questions according to the interview guide, listened carefully, refrained from making suggestions or leading the interviewee, and used reflective questions to allow the interviewee to express their feelings. If necessary, the interviewers provided supplementary descriptions of the concept of home hospice care and repeated and summarized the interviewee’s statements. Within 24 h of the interviews, the recording was converted into text, and the text data were returned to the interviewee for reading to ensure the accuracy of the data.

## 5 Data analysis

### 5.1 Quantitative data analysis

The SPSS version 27.0 was used for descriptive statistics and difference comparisons. Categorical data are represented by frequency (*n*) and percentage (%). Continuous data are expressed as mean ( $\bar{x}$ ) and standard deviation (*SD*). In univariate analysis, comparisons of rates are made using the Chi-square test, and comparisons of means are made using the *t*-test. Multivariate analysis is conducted using logistic regression. Considering that the variable of education has a great OR value in the model, and it is reclassified or removed, primary school and below and junior high school are combined, and the sensitivity analysis is repeated by binary logistic regression. The significance level is set at *α* = 0.05 (two-tailed).

### 5.2 Qualitative data analysis and result integration

Directed content analysis was employed for data analysis. After importing the text into the Nvivo 14.0 software, the Anderson Model was used as the coding framework, and the coding process was carried out in the following steps: (1) repeatedly listening to the recordings and reading the transcribed texts to gain an overall sense; (2) identifying factors influencing the utilization of home hospice care by older adults, which is the open coding; (3) aggregating all codes and, through repeated comparison and categorization, grouping codes with the same attributes into the same category, which is the axial coding; (4) classifying themes into three dimensions: predisposing factors, enabling factors, and need factors, which is the selective coding. Finally, the results were integrated using the parallel data comparison method (34). First, quantitative and qualitative data were placed side by side for direct comparison of their consistencies and differences. The integrated results were then jointly presented in tables to illustrate the findings and meta-inferences. All the processes were performed independently and simultaneously by two researchers. In case of disagreement, our research team held a collective discussion and finally reached a conclusion, which ensured the logic and reliability of each topic.

## 6 Results

### 6.1 Common method bias

Harman single-factor method was used to test the common method bias (35). The results showed that the characteristic roots of the 9 factors were greater than 1, and the explained variation of the first factor was 30.06%, which was lower than the critical value of 40.00%. Therefore, it shows that there is no obvious common method bias in this study.

### 6.2 Quantitative results

The average age of older adults was 70.73 years (*SD* = 10.12), ranging from 60 to 96 years old. A total of 45 (13.55%) older adults utilized home hospice care services. The Home Hospice Care Service Need Scale score was 24–100 (60.67 ± 19.91). The Knowledge, Attitude, and Behavior of Home Hospice Care Scale score was 32–124 (62.37 ± 17.80). Univariate analysis results showed that the utilization of home hospice care by older adults differed significantly in terms of age (*F* = 6.971, *p* = 0.008), type of caregiver (*F* = 30.981, *p* < 0.001), physiological needs scores (*t* = 9.995, *p* < 0.001), psychological needs scores (*t* = 12.901, *p* < 0.001), social needs scores (*t* = 4.069, *p* < 0.001), spiritual needs scores (*t* = 8.971, *p* < 0.001), knowledge scores (*t* = −9.442, *p* < 0.001), belief scores (*t* = −4.315, *p* < 0.001), and attitude scores (*t* = −10.418, *p* < 0.001). The general characteristics of older adults and the results of univariate analysis are presented in Table 1. Furthermore, binary logistic regression analysis was used to examine the associated factors of home hospice care service utilization, with detailed results shown in Table 2.

**Table 1:** Characteristics of participants and analysis of differences in home hospice care acceptance.

Variables	Accept		*χ^2^/t/F*	*P*
	Yes (n(%))	No (n(%))		
Gender				
Male	24(18.05)	109(81.95)	3.819	0.051
Female	21(10.55)	178(89.45)		
Age (years)				
60–69	14(8.14)	158(91.86)	9.543	0.008
70–79	17(21.52)	62(78.48)		
80+	14(17.28)	67(82.72)		
Nationality				
Han	29(12.83)	197(87.17)	0.315	0.574
Minority	16(15.09)	90(84.91)		
Marital status				
Married	33(15.28)	183(84.72)	1.628	0.443
Unmarried/divorced	1(10.00)	9(90.00)		
Widowed	11(10.38)	95(89.62)		
Education				
Primary school and below	6(4.65)	123(95.35)	14.586	<0.001
Middle school	19(17.92)	87(82.08)		
High school and above	20(20.62)	77(79.38)		
Resident				
Rural	11(13.1)	73(86.9)	0.020	0.887
Urban	34(13.71)	214(86.29)		
Monthly income (Yuan)				
<1,000	21(17.36)	100(82.64)	2.352	0.308
1,000–3,000	12(11.54)	92(88.46)		
>3,001	12(11.21)	95(88.79)		
Payment				
Health insurance	42(13.68)	265(86.32)	0.095	0.758
Self-paying	3(20.00)	12(80.00)		
Living form				
With families	36(13.64)	228(86.36)	0.007	0.931
Alone	9(13.24)	59(86.76)		
ADL				
100	30(13.23)	180(86.77)	3.315	0.346
61–99	11(18.64)	55(81.36)		
41–60	3(4.92)	31(95.08)		
≤40	1(26.09)	21(73.91)		
Chronic disease				
No	20(21.51)	73(78.49)	6.971	0.008
Yes	25(10.46)	214(93.45)		
Caregiver				
Family members	32(25.40)	94(74.60)	30.981	<0.001
Nursing workers	11(14.28)	66(85.72)		
Not need	2(1.55)	127(98.45)		
NRS scores	1.09 ± 1.535	1.35 ± 1.507	1.072	0.285
Needs				
Physiological	11.89 ± 4.92	20.35 ± 7.14	−9.995	<0.001
Psychology	5.84 ± 2.70	12.39 ± 5.35	−12.901	<0.001
Society	13.56 ± 4.42	16.76 ± 7.26	−4.069	<0.001
Spiritual	9.84 ± 2.76	14.24 ± 4.49	−8.971	<0.001
K-A-P				
Knowledge	23.56 ± 5.38	16.01 ± 4.92	9.442	<0.001
Attitude	32.11 ± 10.40	25.17 ± 7.27	4.315	<0.001
Practice	26.78 ± 6.59	18.05 ± 5.70	10.418	<0.001

ADL, the activities of daily living (ADL) score; NRS, score of NRS 2002.

**Table 2:** Factors of influencing accepting home hospice care among the terminally older adults.

Variables	*B*	*SE*	Wald *χ*^2^	*P*	*OR*	95%CI	
						LLC	ULC
Age (years)			7.278	0.026			
70–79	2.332	1.161	4.035	0.045	10.297	1.058	100.193
80+	2.829	1.098	6.638	0.010	16.920	1.968	145.503
Education			7.697	0.021			
Middle	4.016	1.511	7.061	0.008	55.455	2.869	1,072.056
Senior and above	2.043	1.429	2.043	0.153	7.716	0.468	127.079
Chronic disease	−2.720	1.029	6.993	0.008	0.066	0.009	0.495
Caregiver			11.735	0.003			
Nursing worker	0.398	0.990	0.162	0.688	1.489	0.214	10.356
Not need	−5.723	1.675	11.676	0.001	0.003	0.001	0.087
Needs							
Physiological	−0.236	0.097	5.968	0.015	0.790	0.654	0.954
Psychology	−0.999	0.267	13.994	<0.001	0.368	0.218	0.622
Society	0.532	0.162	10.812	0.001	1.703	1.240	2.339
Spiritual	−0.408	0.142	8.234	0.004	0.665	0.503	0.879
K-A-P							
Knowledge	0.439	0.130	11.454	0.001	1.551	1.203	2.000
Attitude	−0.155	0.077	4.069	0.054	0.857	0.737	0.996
Practice	0.148	0.099	2.258	0.133	1.160	0.956	1.407
Constant	−2.627	2.347	1.253	0.263	0.072		

*R*^2^ = 0.479, *χ*^2^ = 47.316, *p* = 0.874. Variables coding: age: 60–69 = 1, 70–79 = 2, 80+ = 3; education: primary school and below = 1, middle school = 2, high school and above = 3; chronic disease: no = 0, yes = 1; caregivers: family members = 1, nursing workers = 2, not need = 3.

SE, standard error; K-A-P, Knowledge, Attitude, and Practice scores; LLC, lower limit of confidence interval; ULC, upper limit of confidence interval.

### 6.3 Sensitivity analysis

After merging the variable of education, the analysis showed that age, education, chronic disease, caregiver, physiological needs, psychological needs, social needs, spiritual needs, and knowledge, attitude, and practice are factors associated with utilization (*P*<0.05). When removed, the same results are shown, indicating that the results are robust (see Table 3).

**Table 3:** Factors of influencing accepting home hospice care among the terminally older adults.

Variables	*B*	*SE*	Wald *χ*^2^	*P*	*OR*	95%CI	
						*LLC*	*ULC*
Age (years)			6.84	0.033			
70–79	2.817	1.137	6.136	0.013	16.721	1.8	155.307
80+	2.232	1.06	4.436	0.035	9.32	1.168	74.394
Education							
Senior and above	−0.419	0.881	0.226	0.635	0.658	0.117	3.7
Chronic disease	−1.876	0.905	4.3	0.038	0.153	0.026	0.902
Caregiver			8.761	0.013			
Nursing worker	−0.131	0.981	0.018	0.894	0.877	0.128	6.003
Not need	−4.5	1.526	8.699	0.003	0.011	0.001	0.221
Needs							
Physiological	−0.213	0.093	5.223	0.022	0.808	0.673	0.97
Psychology	−0.854	0.22	15.093	0	0.426	0.277	0.655
Society	0.505	0.146	11.98	0.001	1.657	1.245	2.205
Spiritual	−0.408	0.145	7.969	0.005	0.665	0.501	0.883
K-A-P							
Knowledge	0.316	0.104	9.226	0.002	1.371	1.119	1.681
Attitude	−0.216	0.075	8.255	0.004	0.806	0.695	0.934
Practice	0.264	0.107	6.045	0.014	1.302	1.055	1.607
Constant	0.04	2.163	0	0.985	1.041		

*R*^2^ = 0.475, *χ^2^* = 51.941, *P* = 0.874. Variables coding: age: 60–69 = 1, 70–79 = 2, 80+ = 3; education: middle school and below = 1, high school and above = 2; chronic disease: no = 0, yes = 1; caregivers: family members = 1, nursing workers = 2, not need = 3.

SE, standard error; K-A-P, Knowledge, Attitude, and Practice scores; LLC, lower limit of confidence interval; ULC, upper limit of confidence interval.

### 6.4 Qualitative research results

A total of 16 individuals participated in semi-structured interviews, with 13 (81.25%) utilizing home hospice care and three receiving treatment in the hospital, with an average age of 68.50 years (*SD* = 6.36). All were cancer patients, and their detailed characteristics are shown in Table 4. From the interviews, 29 factors influencing the utilization of home hospice care by terminally ill older individuals were identified. These associated factors were first categorized into physiological needs, psychological needs, social needs, knowledge, beliefs, and attitudes, and then all categories were grouped into the themes of predisposing factors, enabling factors, and need factors within the Andersen Model. Detailed information is presented in Table 5. The integrated results are detailed in Table 6.

**Table 4:** Demographic characteristics of the participants (*n* = 24).

No.	Gender	Age	Duration (month)	Education	Residence	Income
N1	Male	68	/	High school	Rural	1
N2	Male	63	/	Junior school	Rural	1
N3	Male	86	1	High school	Urban	3
N4	Male	70	1	Junior school	Urban	3
N5	Female	66	2	Junior school	Urban	2
N6	Male	60	3	Junior school	Rural	1
N7	Female	68	1	University	Urban	2
N8	Male	62	1	Junior school	Urban	2
P9	Male	69	1	Primary school	Urban	1
N10	Female	72	1	High school	Urban	2
N11	Male	61	/	Junior school	Rural	1
N12	Male	74	1	University	Urban	3
N13	Male	72	2	Junior school	Urban	2
N14	Female	65	1	Primary school	Rural	2
N15	Male	67	2	High school	Urban	2
N16	Male	73	3	Primary school	Urban	1

/: have not received home hospice care; income 1: <1,000 Yuan/Month; income 2: (1000–3,000) Yuan/Month; income 3:>3,000 Yuan/Month.

**Table 5:** Mapping of facilitators and barriers to the Anderson domains.

Anderson	Dimension	Themes	Representative quotations
Prerequisite factors	Knowledge	Live better at home (+)	N13: Staying at home, I would live better.
		Save resources (+)	N1: It can be said that continuing to stay in hospitals is a waste of national resources.
		Informed by doctors or relatives (+)	N3: I do not know about hospice, but my daughter does. N8/N13: The doctors told me there was home hospice.
		Recognized not need for treatment (+)	N12: I did not believe that continuing therapy would work, so I refused to continue therapy and went home. N13: The doctor told me, and I understand that there’s no point in any further treatment. N16: I realized I was dying, so I stopped chemo and stayed at home.
	Attitude	Awareness and acceptance of dying (+)	N3: Maybe I do not have much time left. I’d be better off at home. N13: I knew I am sick and dying.
		Doctors and nurses will visit at home (+)	N14: The doctors and nurses will visit me at home, which is much easier.
		Accept death calmly (+)	N3: I’m open-minded enough to look at death in peace, and when I die, just scatter my ashes at sea. N6: They (doctors) told me I did not have long to live, and I was coming to terms with it.
		Deny dying (−)	N1: I do not like to talk about it (death and home hospice care). N2: I was told by doctors, but I do not think I’m not yet (dying).
	Practice	Follow doctor’s advice (+)	N12: We mainly followed the doctor’s advice and stay at home, and usually did not take the initiative to ask for hospitalization.
		Follow the son ‘s decision (+)	N7: The choice to stay at home was basically the son’s decision.
Enabling factors	Service resource	Convenient service (+)	N5: I think the service is good now and it’s convenient to drive here once in a while. N9: Home hospice care is much better than before, after all, we did not have to go to the hospital all the time.
		Long distance from the service point (−)	N1: My home is about 20 km from the service point, and even a phone call at any time cannot make up for the distance. N4: It’s best to stay at home, but it’s too far away.
		Short distance from the service point (+)	N7: It’s easy to pick up the painkillers after you take them, which is a 3 to 4 min drive. N15: The doctor advised me to take home hospice service because my home is not far from the service point, and I accepted.
		Low accessibility (−)	N1: If only this kind of service could be popularized in towns and villages. N11: I do not consider home hospice services, which are lacking mainly in rural areas
	Economics	Give up treatment due to economically insufficient (+)	N11: If the money is spent on treatment, we’ll go home. N14: I wanted to give up and go home because the treatment was too expensive.
		Financial burden (+)	N11: Although my two children share the cost of my treatment, the financial pressure is still heavy. N12: Although we have a monthly income of 8, 000 yuan, the cost of treatment is so large that we still have to keep the money for basic living.
		Low income (+)	N1: I am a rural person with a low income and cannot afford the expensive treatment. N9: My son is the only one who works for money, so I have to accept home hospice services.
	Caregivers	Lack of caregivers (−)	N9: I’m not being cared for enough at home because we do not have enough people. N11: I had to stay at the hospital because my children had to work and could not take care of me.
	Relatives’ feelings	Sense of guilt (+)	N1: My children are regretting paying little attention to my health and are now actively supporting my treatment.
Needs factors	Physiological	Meet pain relief needs (+)	N1: Because the pain is too intense to be relieved at home, you must come to the hospital for an intravenous drip. N7: It’s actually better to manage pain at home. N11: My main symptom is pain. I would feel ok when that’s relief.
		Immediately pain relief needs (−)	N1: Pain could be relieved in time staying in the hospital. N11: Sometimes it’s too late, and the pain lasts a long time.
		Limited needs (+)	N10: Give me painkillers, and I’m content enough. N13: When the pain relief is achieved, we feel satisfied. N16: I do not really need anything other than pain relief.
		Sufficient self-care ability (+)	N3: I think I can take care of myself, so I might as well stay at home. N12: I chose to live at home mainly because I can still take care of myself.
	Society	Need for social communication (+)	N3: He′ll feel better when he’s at home and can hang out with colleagues and friends. N10: When I talked to my patient and learned that she had given up treatment, I would staying home and waiting to die.
		Concerns about disease privacy known by neighbors (−)	N5: It’s not good for the neighbors to know about my illness. N11: If neighbors know I am sick, they will talk about my illness.
	Psychology	Heavy disease-related psychological burden (−)	N1: The psychological burden is heavy, and I cannot sleep well all night. So it’s better to be at home than in a hospital. I worry if the medical staff is sufficient to provide home care when I am at home.
		Feel relax when with families or at home (+)	N3: I’m more relaxed when staying at home. N11: There are other family members to help me ease the mood when staying at home, but I must face it alone when in hospital.

**Table 6:** Combine and comparison between the quantitative and qualitative findings.

Anderson	Contents	Quantitative findings	Qualitative findings	Meta-inferences
Predisposing factors	Knowledge	Knowledge scores (*B* = 0.439, *p* = 0.001)	Informed by doctors or relatives (+)	Confirm: People with more knowledge of home hospice care are more likely to accept home hospice care. Extension: Healthcare staff and relatives can help terminal older adults learn about home hospice care. When older individuals perceive end-stage care as a misuse of medical resources, home hospice care can provide a higher quality of life in their final stage. Extension: Qualitative studies provide additional insights. It showed that accept terminal state and positive attitudes toward death had a positive effect on receiving services.
			Live better at home (+)	
			Save resources (+)	
			Recognized the need for treatment (+)	
	Attitude	Attitude scores (*B* = −0.155, *p* = 0.054)	Awareness and acceptance of dying (+)	Extension: The results were supplemented by qualitative studies. Older individuals tend to follow the advice of doctors and family members when making healthcare decisions.
			Recognize the role of home hospice care (+)	
			Accept death calmly (+)	
			Deny dying (−)	
	Practice	Practice scores (*B* = 0.148, *p* = 0.133)	Follow doctor’s advice (+)	
			Follow the son ‘s decision (+)	
Enabling factors	Economics	Monthly income (χ^2^ = 2.352, *p* = 0.308)	Give up treatment due to economically insufficient (+)	Extension: Qualitative findings add insights. Economic factors require a comprehensive assessment of income and expenditure. When older adults perceive the financial burden, they are more inclined to accept services.
			Financial burden (+)	
			Low income (+)	
	Caregivers	Not need (family members = 0) (*B* = −5.723, *P* = 0.001)	Lack of caregivers (−)	Confirm: When there are sufficient caregivers, older people at the end of life tend to receive services.
	Service resource	–	Convenient service (+)	Extension: Qualitative studies provide evidence of older adults’ concerns about home hospice resources. The availability, accessibility and convenience of the resource would be taken considered by older adults.
			Long distance from the service point (−)	
			Short distance from the service point (+)	
			Low accessibility (−)	
Need factors	Physiological needs	Physiological scores (*B* = −0.236, *p* = 0.015)	Meet pain relief needs (+)	Confirm: older adults people with little physiological needs are more likely to use home hospice services. Extension: Relieving pain symptoms is a basic need for older adults at home.
			Pain relief timely needs (−)	
			Limited needs (+)	
			Sufficient self-care ability (+)	
	Social needs	Social needs (*B* = 0.532, *P* = 0.001)	Need for social communication (+)	Confirm: Both qualitative and quantitative studies showed that social support positively affected the use of home hospice services by older people.
			Concerns about disease privacy known by neighbors (−)	Extension: Qualitative studies provide additional insight that stigma can prevent the older adults from accepting home hospice services.
	Psychological needs	Psychological needs (*B* = −0.999, *P*<0.001)	Heavy disease-related psychological burden (−)	Extension: The results were complemented by the quantitative and qualitative studies. When the sense of security is not satisfied, older adults tend to be hospitalized. When family members can provide psychological support for older adults, older adults tend to receive hospice services at home.
			Feel relax when with families or at home (+)	

## 7 Discussion

### 7.1 Predisposing factors associated with the utilization of home hospice care by the older adults

When exploring the predisposing factors for the utilization of home hospice care services among older adults, first, age is a factor that influences the use of home hospice care, which is similar to the findings of Kumar et al. (18). In our study, older people are more likely to choose home-based hospice care services, which may be related to their more ingrained ‘bottom-up’ concept and greater acceptance of death. Some researches also show that the proportion of older adults who chose to die at home is the highest (14.5 times that of hospitals, 3.4 times that of nursing institutions) (36), and their acceptance of death scores are also higher (37). Second, older adults with chronic diseases are also more inclined to utilize home hospice care services. This might be because these individuals have endured economic burdens for a long time, and home care is usually less expensive than hospitalization (38). However, qualitative research indicates that the factors are complex, including self-care ability and the pursuit of quality of life, which are relatively consistent with the existing research results (39, 40). In the future, methods such as longitudinal studies should be adopted to conduct more in-depth research on the utilization of palliative care services by older people in order to better understand the interaction of these factors and their impact on the utilization of services. More importantly, both quantitative and qualitative studies indicated that awareness and knowledge about home hospice care services were key factors in utilization. This factor positively influences the utilization behavior of older adults, which fully demonstrates the particular importance of doing well in promoting home hospice care. The qualitative study results supplement that older adults learn about home hospice care through doctors or families, with the role of doctors’ notification being more prominent, which is consistent with the research results of Lane et al. (41). Therefore, in the background of the cultural of taboos around death, promoting hospice care through doctors or families might be more significantly effective. In addition, the level of knowledge about home hospice care services (score status) also positively affects utilization behavior. The integrated results indicated that, in terms of knowledge about home hospice care, the subjective factors for older adults to utilize it were mainly recognizing that it can improve the quality of life and save medical resources. This finding reflects the terminally ill older adults’ pursuit of quality of life and less economic burden. However, old adults in the UK and the US are more aware of hospice care at home than in China, as they have raised public awareness of hospice care through extensive publicity and education (42). Therefore, to improve the acceptance of the service, enhance the awareness of home-based palliative care among the old adults and their families through community education, emphasize its advantages and economy would be advice in the future.

### 7.2 Enabling factors associated with the utilization of home hospice care by the older adults

First, who do not require caregivers is the enabling factor for older adults to use home-based hospice services, which is slightly different from the results in western countries where family caregivers are more willing to choose home-based services (43). The reason might be that the supply is insufficient in China, and most of the pressure of daily care is still borne by family caregivers, which is a huge caring burden when terminally ill older adults stay at home (44). Generally, the terminally older are also concerned about the quality and safety of care services (45). Therefore, it is necessary to strengthen the training of nursing staff, enrich nursing human resources, such as collaborative training between medical institutions and society, and increase efforts to train medical caregivers and older adult caregivers. In fact, the decision-making of families and the dying older is a complex issue that might be emotionally contradictory (46). Qualitative research shows that when families feel guilty due to lack of care, they tend to choose hospitalization with the intention of making up for the sense of guilt, as they might think that choosing hospice care is equivalent to giving up treatment (47). Therefore, it is also necessary to improve the understanding of relatives on death and hospice care. In the future, various forms of community education activities should be carried out, such as lectures, workshops, and brochures, to improve the awareness of the population. Third, a huge economic burden is an incentive, and evidence also shows that hospice services can reduce the cost of treatment (48). It is worth noting that assessing the specific income does not accurately predict the motivation for utilization. In the future, the subjective economic burden and objective income of the older and their families should be assessed at the same time. It is necessary to learn from some developed countries, such as South Korea and the UK, which provide financial support for the health services of hospice care and clarify the medical insurance reimbursement system (49). Additionally, home-based hospice care resources are an important motivational factor. In qualitative studies, older adults have indicated that the long distance between service locations and the lack of widespread resources are reasons they consider not to use home-based services. Terminally ill patients face a multitude of painful symptoms (50), especially those in the late stages of cancer. When resources to alleviate pain are not readily available, they might choose to be hospitalized. Therefore, attention should be paid to perfecting the rational allocation of distance and resources for home-based services by centering on community health services, with the main guidance from hospitals and auxiliary services from volunteers.

### 7.3 Demand factors associated with the utilization of home hospice care by the older adults

Interestingly, quantitative research has found that the higher the physiological and psychological needs, the less inclined they were to utilize home-based services. Qualitative research indicated that older adults did not have many needs for home-based services, which are urgent for pain relief. Home-based services are limited in China, with visits ranging from one time a month to one time a week, which hinders their ability to provide timely pain relief and a sense of security when symptoms arise (51). Therefore, home-based services are a good option when the demand is not high for older adults. Next, qualitative research findings further indicate that even within the context of limited services, family members’ psychological support is a facilitating factor for utilization. In the future, a comprehensive assessment of the varying degrees of needs of older adults, such as the most urgent, urgent, and general, should be conducted to first meet the most pressing needs and guide families in psychological nursing skills, making resource allocation more rational and service development feasible. However, when there is a high level of social needs, the likelihood of older adults utilizing them increases, and they also express a need for community interaction. Contrary to social demands, they have indicated that the negative attitudes of neighbors toward the end of life can hinder utilization behavior. Studies have shown (52) that disease stigma can lead to a sense of shame and social alienation in older adults. Participating in community activities and receiving support from the community can increase the happiness of older adults and reduce their feelings of loneliness. It also indicates that community support involving medical personnel can extend the time that older adult individuals spend at home. Therefore, it is possible to provide support for home-based older adult care through community death education and the establishment of informal organizations that include medical staff.

## 8 Limitations

The following are the limitations of our study. The older adults who utilized home hospice care in this study were mostly patients in the late stages of cancer, and the results may not be as representative of other terminally ill older adult individuals. Second, the study was conducted in a less economically developed province in western China, and the economic factors may differ from other regions. Therefore, future studies could expand the range of diseases and regions under investigation, employing stratified sampling based on disease types and regional economies to make the study results more representative. Third, this study is a cross-sectional survey and cannot reflect the psychological factors at different stages of terminally ill patients. Future longitudinal studies could explore the utilization trends and associated factors of home hospice care. Fourth, due to the need for patients to recall and the influence of patients’ social expectations, there is a certain recall bias and social expectation bias in the study. Finally, this cross-sectional study cannot establish a causal relationship.

## 9 Conclusion

In comparison with South Korea and China, the utilization rate in this study is only 13.55%, which fully demonstrates the gap between intention and acceptance. This further illustrates the significant importance of understanding the influencing factors. The factors influencing the utilization of home hospice care services by older adults are complex. The utilization of home-based services is influenced by both objective conditions and subjective intentions. Our study, guided by Andersen’s Health Services Utilization Model, employs mixed methods to explore the potential factors in the decision-making of terminally ill older individuals regarding home-based hospice care. Ultimately, the positive enabling factors for utilization by older adults are identified as advanced age, chronic illness, and good knowledge of it. The main positive enabling factors are caregivers being nursing staff, a significant perceived economic burden, and accessible care resources, while the negative factor is the emotional involvement of families. Positive needs factors include social needs, and negative ones include physical and psychological needs. These results provide a comprehensive framework for understanding the facilitators and barriers to the utilization of home-based hospice care services by terminally ill older adults. In the future, intervention strategies to increase the utilization rate of home-based hospice care for older adults can be formulated based on these associated factors.

## Data Availability

The datasets presented in this study can be found in online repositories. The names of the repository/repositories and accession number(s) can be found below: the materials in this article are from the author’s original research, and further inquiries can be directed to the corresponding author.
