# Supplementary material for: Associated factors of home hospice care utilization by the terminally ill older adults: a mixed-methods study
Source: Front Public Health. 2025 Jun 18;13:1519712. doi: 10.3389/fpubh.2025.1519712 (PMC12213468; doi:10.3389/fpubh.2025.1519712)
Supplement: Supplementary file 1 [file Supplementary_file_1.docx]

**Appendix 1**

**Home hospice Care Service Need Scale**

**1 Physiological needs**

- 1. Need daily life care ( such as laundry, cleaning, etc. )
  2. Need guidance on daily activities and exercise.
  3. Need to reduce physical discomfort ( such as pain, vomiting, etc. )
  4. It is necessary for medical staff to evaluate and observe somatic symptoms.
  5. It is necessary for medical staff to provide nursing technical guidance ( such as oxygen inhalation, dressing change, etc. )
  6. It is necessary to avoid accidents ( such as falls, suffocation, etc. )

**2 Psychological needs**

2. 1 Need to be respected by others.

2. 2 Need to fully express the inner demands and feelings.

2. 3 Need to relieve anxiety, depression emotions.

2. 4 Need to get professional psychological counseling.

**3 Social needs**

3. 1 Need to understand the disease, symptoms related knowledge.

3. 2 Need to understand their own condition changes at any time.

3. 3 Need to communicate with others.

3. 4 Need to join the patient group, peer support each other.

3. 5 Need someone else to provide temporary care.

3. 6 Need others to provide long-term companionship.

3. 7 Need to get care and care from social groups.

**4 Spiritual needs**

4. 1 Need to appreciate beautiful things ( such as food, beautiful scenery, etc. )

4. 2 Need to fulfill unfulfilled desires.

4. 3 Education in death is needed.

4. 4 Matters after death need to be discussed.

**Appendix 2**

**Knowledge-Attitude-Practice Scale for Home Hospice Care**

The scale includes three dimensions: knowledge (The individual’s awareness of home hospice care, 8 items), attitude (The individual’s views and feelings on home hospice care, 12 items), and practice (The individual’s behavior related to home hospice care, 9 items), with a total of 29 items.

**1 Knowledge dimension**

- 1. Home hospice care does not pursue curative treatment.
  2. Home hospice care service is a supportive medical measure for patients and relatives at the end of life to provide medical care, nursing, psychological counseling, and death education at home.
  3. Home hospice care services can reduce the burden of family and society and save medical resources.
  4. It is not only the patients who are getting worse that need home hospice care.
  5. Home hospice care can control pain and relieve other physical symptoms.
  6. Home hospice care maintains the dignity of older adults at the end of their life.
  7. Home hospice care can prevent and alleviate the anxiety of older adults at the end of life.
  8. Home hospice care can help older adults to accept death calmly.

**2 Attitude dimension**

2. 1 I think home hospice care is meaningful.

2. 2 I think at the end of the disease, the quality of life is more important than the length of life.

2. 3 I think it is normal to discuss the issue of death.

2. 4 I think home hospice care does not mean giving up the life of older adults.

2. 5 I believe that the current hospice care model of hospitals and hospice care institutions fails to meet the physical and mental needs of patients at the end of life.

2. 6 I hope I can get supportive treatment at home from a professional.

2. 7 I think it is necessary to popularize home hospice care services.

2. 8 I wish I could die at home, not in an unfamiliar hospital room.

2. 9 I think my physical condition is more suitable for home hospice care.

2. 10 I don 't want to burden my family with unnecessary treatment at the end of my life.

2. 11 I think it 's important to have family or friends at the end of life.

2. 12 When making decisions on home hospice care, I hope that professionals can give me objective advice.

**3 Practice dimension**

3. 1 I have paid attention to home hospice care.

3. 2 If there are lectures on home hospice care, I will take the initiative to attend.

3. 3 When my disease cannot be cured, I will choose home hospice care.

3. 4 I think it is normal to discuss the issue of death.

3. 5 I believe that the current hospice care model of hospitals and hospice care institutions fails to meet the physical and mental needs of patients at the end of life.

3. 6 I believe that the current hospice care model of hospitals and hospice care institutions fails to meet the physical and mental needs of patients at the end of life.

3. 7 At the end of life, I will take the initiative to express my physical and psychological needs with my family or home hospice care personnel.

3. 8 If friends or relatives around receive home hospice care, I express respect and support.

3. 9 Home hospice care does not conflict with my philosophy, beliefs and local customs.
